# Diagnostic accuracy of MRI for evaluating myometrial invasion in endometrial cancer: *a comparison of MUSE-DWI, rFOV-DWI, and DCE-MRI*

**DOI:** 10.1007/s11547-023-01635-4

**Published:** 2023-04-29

**Authors:** Takashi Ota, Takahiro Tsuboyama, Hiromitsu Onishi, Atsushi Nakamoto, Hideyuki Fukui, Keigo Yano, Toru Honda, Kengo Kiso, Mitsuaki Tatsumi, Noriyuki Tomiyama

**Affiliations:** Department of Diagnostic and Interventional Radiology, Osaka University Graduate School of Medicine, D1, 2-2, Yamadaoka, Suita, Osaka 565-0871 Japan

**Keywords:** Diffusion magnetic resonance imaging, Uterine neoplasms, Endometrial neoplasms, Neoplasm staging, Diffusion-weighted imaging

## Abstract

**Objectives:**

To compare the image quality of high-resolution diffusion-weighted imaging (DWI) using multiplexed sensitivity encoding (MUSE) versus reduced field-of-view (rFOV) techniques in endometrial cancer (EC) and to compare the diagnostic performance of these techniques with that of dynamic contrast-enhanced (DCE) MRI for assessing myometrial invasion of EC.

**Methods:**

MUSE-DWI and rFOV-DWI were obtained preoperatively in 58 women with EC. Three radiologists assessed the image quality of MUSE-DWI and rFOV-DWI. For 55 women who underwent DCE-MRI, the same radiologists assessed the superficial and deep myometrial invasion using MUSE-DWI, rFOV-DWI, and DCE-MRI. Qualitative scores were compared using the Wilcoxon signed-rank test. Receiver operating characteristic analysis was performed to compare the diagnostic performance.

**Results:**

Artifacts, sharpness, lesion conspicuity, and overall quality were significantly better with MUSE-DWI than with rFOV-DWI (*p* < 0.05). The area under the curve (AUC) of MUSE-DWI, rFOV-DWI, and DCE-MRI for the assessment of myometrial invasion were not significantly different except for significantly higher AUC of MUSE-DWI than that of DCE-MRI for superficial myometrial invasion (0.76 for MUSE-DWI and 0.64 for DCE-MRI, *p* = 0.049) and for deep myometrial invasion (0.92 for MUSE-DWI and 0.80 for DCE-MRI, *p* = 0.022) in one observer, and that of rFOV-DWI for deep myometrial invasion in another observer (0.96 for MUSE-DWI and 0.89 for rFOV-MRI, *p* = 0.048).

**Conclusion:**

MUSE-DWI exhibits better image quality than rFOV-DWI. MUSE-DWI and rFOV-DWI shows almost equivalent diagnostic performance compared to DCE-MRI for assessing superficial and deep myometrial invasion in EC although MUSE-DWI may be helpful for some radiologists.

## Introduction

MRI is an essential diagnostic modality for the assessment of uterine cancers [1–3]. Conventionally, the combination of T2-weighted imaging (T2WI) and dynamic contrast-enhanced (DCE) imaging has been accepted as providing accurate staging for endometrial cancers (ECs), and as for DCE-MRI, myometrial invasion of the tumor is best depicted at the equilibrium phase (approximately 2 min after the contrast injection) [4]. Recent meta-analyses have indicated that diffusion-weighted imaging (DWI) may be an alternative to DCE-MRI in preoperative staging of EC [5, 6]. Moreover, it has been reported that the simultaneous use of T2WI, DCE-MRI, and DWI provides the highest sensitivity and specificity for detecting deep myometrial invasion [7]. Accordingly, DWI appears to show promise for application to EC staging. Furthermore, radiomics analysis with multi-sequence MRI was reported to be useful in predicting the microsatellite instability of EC genes which has been proved to be an important prognostic factor recently [8].

Lymph node metastasis, which is the strongest predictor of recurrence, is related to deep myometrial invasion (≥ 50% of myometrial depth). Therefore, lymphadenectomy can be considered for intermediate or high-risk EC (grade 3 and/or deep myometrial invasion), but it is not recommended for low-risk EC (grade 1 or 2 without deep myometrial invasion) [9]. Accordingly, preoperative assessment of myometrial invasion by MRI is crucial in patient management and for tailoring the surgical approach.

Conventional DWI (cDWI) uses echo-planar imaging (EPI) because of its rapid scan time and minimal artifacts from respiratory and cardiac motion [10]. However, EPI is prone to geometric distortion, and spatial resolution cannot be increased. Therefore, EPI has relatively low spatial resolution [11].

There has recently been remarkable progress in DWI. Newly developed distortion reduction technology has enabled high-spatial-resolution imaging, including a reduced field-of-view (rFOV) technique [12]. To achieve high-resolution imaging with decreased susceptibility artifacts, this technique uses two-dimensional, spatially selective echo-planar radiofrequency excitation pulse for limited excitation in the phase-encoding direction, and the required number of k-space lines in the phase-encoding direction is decreased by FOV reduction [12]. A previous study reported that the rFOV technique yielded better assessment of myometrial invasion in EC compared to cDWI [13]; however, the rFOV technique has a disadvantage of a small FOV [13]. Therefore, to improve the image interpretation in EC staging, it is necessary to increase the FOV while maintaining the spatial resolution.

Multiplexed sensitivity encoding (MUSE) is another recently developed technique [14]. Multi-shot EPI enables distortion reduction to achieve high-spatial-resolution imaging; however, shot-to-shot phase variations can severely degrade the image quality [15]. With MUSE-DWI, a phase navigator is used for each segment, and the phase navigator and parallel imaging are used to solve motion-induced phase errors [16].

We have two choices for high-resolution DWI in the female pelvis: MUSE-DWI and rFOV-DWI. So far, no study has reported a comparison of these techniques. This retrospective study aimed to compare the image quality of MUSE-DWI versus rFOV-DWI and to compare the diagnostic performance of these techniques for evaluating the depth of myometrial invasion with that of DCE-MRI by using the pathological diagnosis as the reference standard.

## Materials and methods

### Patient population

**Fig. 1:**
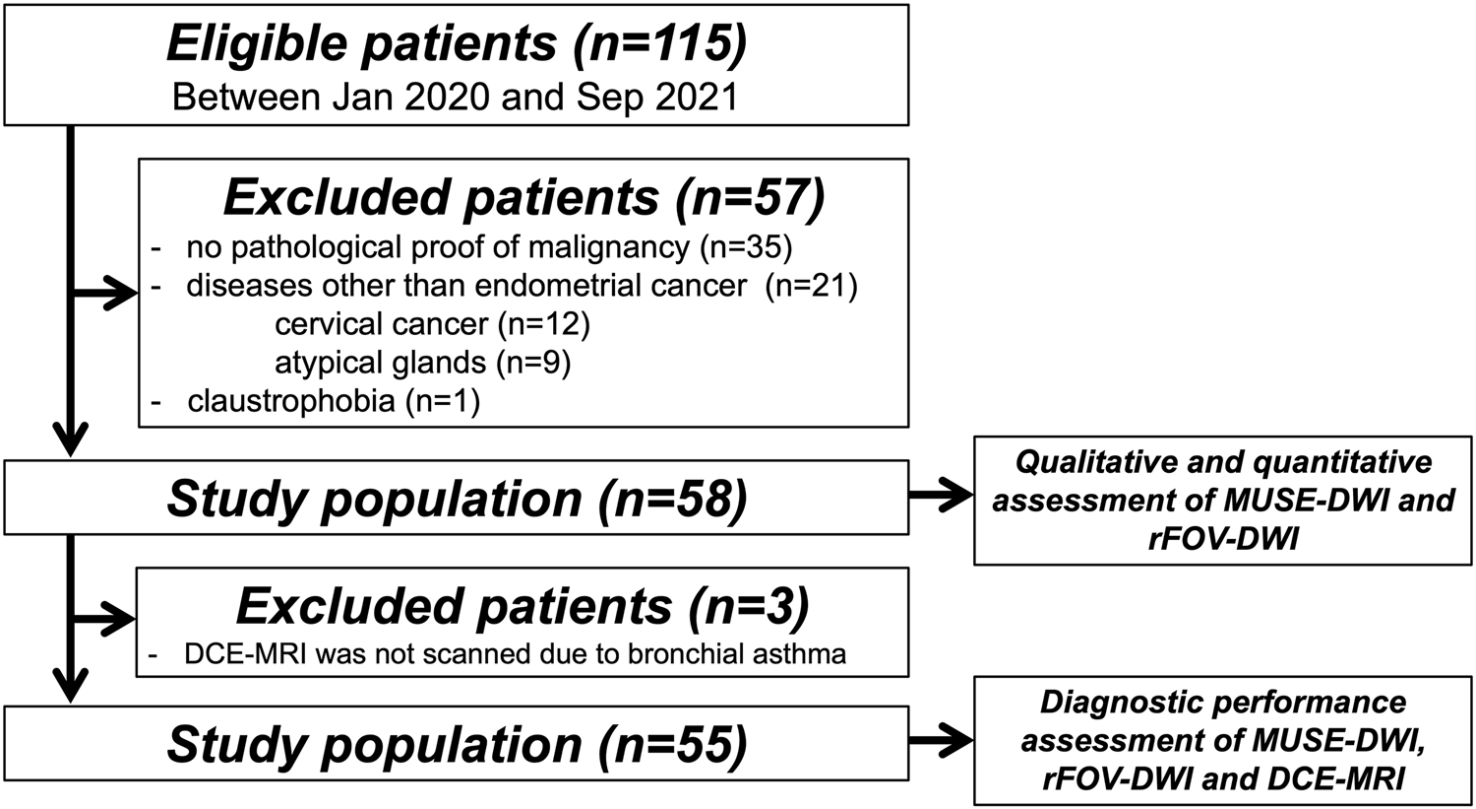
Flowchart of patient enrollment

This retrospective study was approved by the institutional review board of our hospital, which waived requirement for informed consent. Between January 2020 and September 2021, 115 consecutive women who underwent preoperative MR imaging at our hospital because of suspected EC and who had no previous treatment history were enrolled. The exclusion criteria were as follows: (1) no pathological proof of malignancy (*n* = 35); (2) diseases other than EC (cervical cancer, *n* = 12; atypical glands, *n* = 9); and (3) claustrophobia (*n* = 1). The final population comprised 58 patients (mean age, 58.9 ± 9.9 [range, 38–81] years) (Fig. 1). All patients underwent hysterectomy, at a mean of 29.5 ± 14.0 (range, 3–62) days after MR examination. We evaluated qualitative and quantitative assessment of MUSE-DWI and rFOV-DWI in these population. Of the 58 patients, DCE-MRI was not scanned in 3 patients due to bronchial asthma. Thus, we evaluated the diagnostic performance of MUSE-DWI, rFOV-DWI, and DCE-MRI in 55 patients (Fig. 1).

### MR examination

All MR images were acquired using 3.0-T system (Signa Architect, GE Healthcare, Milwaukee, WI, USA) with a 30-channel adaptive imaging receive coil. Unless contraindicated, patients were administered 20 mg scopolamine butylbromide (Nipro, Osaka, Japan) intramuscularly to reduce bowel motion before image acquisition. All patients were scanned in supine position. The MR protocol included T1- and T2WI, rFOV-DWI, MUSE-DWI, and DCE-MRI.

T2WI were acquired in parasagittal (i.e., parallel to the uterine longitudinal axis) and para-axial (orthogonal to the longitudinal axis) planes, using the following parameters: repetition time (TR)/ echo time (TE), 6700–8000/80 ms; slice thickness, 4 mm; slice spacing, 0 mm; flip angle, 90°; FOV, 200 × 200 mm; matrix, 512 × 512; number of excitations (NEX), 2–3; and bandwidth, 41.67 kHz.rFOV-DWI was obtained in parasagittal and para-axial planes with *b*-values of 0 and 1000 s/mm^2^. 2D RF excitation pulse and 180° refocusing pulse were used to reduce the FOV in the phase-encode direction while simultaneously suppressing signal from fat. The scan parameters were as follows: TR/TE, 4500/66.2 ms; slice thickness, 4 mm; slice spacing, 0 mm; flip angle, 90°; FOV, 110 × 70 mm; matrix, 66 × 44; NEX, 10; bandwidth, 166.7 kHz; and acquisition time, 3 min, 9 s. In-plane spatial resolution was 1.67 × 1.59 mm^2^.

MUSE-DWI was acquired in parasagittal and para-axial planes with *b*-values of 0 and 1000 s/mm^2^. MUSE-DWI splits the single-shot EPI into three shots to reduce distortion. The scan parameters were as follows: TR/TE, 6500/78.7 ms; slice thickness, 4 mm; slice spacing, 0 mm; flip angle, 90°; FOV, 240 × 240 mm; matrix, 144 × 144; NEX, 4; bandwidth, 250 kHz; and acquisition time, 4 min, 33 s. In-plane spatial resolution was 1.67 × 1.67 mm^2^.

DCE-MRI was acquired with parasagittal 3D T1WI, and equilibrium phase was obtained with parasagittal and para-axial liver acquisition with volume acceleration (LAVA) in 2 min after contrast material injection. The scan parameters were as follows; TR-TE, 8.3/2.5 ms; slice thickness, 4 mm; slice spacing, 2 mm; flip angle, 12°; FOV, 200 × 200 mm (parasagittal plane), 256 × 140; matrix, 320 × 192 (parasagittal plane), 256 × 140; NEX, 1; bandwidth, 62.4 kHz; and acquisition time, 26 s (parasagittal plane) and 29 s (para-axial plane). In-plane spatial resolution were 0.8 × 1.04 mm^2^ and 0.78 × 1.43 mm^2^, respectively.

### Qualitative image analysis

All MR images were anonymized and transferred to an image viewer (SYNAPSE VINCENT; FUJIFILM, Tokyo, Japan). Three radiologists (with 17 and 10, and 6 years of experience in abdominal radiology) independently performed qualitative visual assessment of rFOV and MUSE-DWI on *b* = 1000 s/mm^2^ images, in terms of artifacts, noise, sharpness, lesion conspicuity, and overall quality. Each item was scored using a 5-point Likert scale, as described in a previous study (Artifact: 1, non-diagnostic; 2, substantial impact on diagnosis; 3, moderate impact on diagnosis; 4, little impact on diagnosis; 5, no artifact; Noise: 1, non-diagnostic; 2, substantial impact on diagnosis; 3, moderate impact on diagnosis; 4, little impact on diagnosis; 5, no impact on diagnosis; Sharpness: 1, non-diagnostic; 2, not sharp; 3, a little sharp; 4, moderately sharp; 5, satisfying sharp; Lesion conspicuity: 1, lesion unidentifiable; 2, no differentiation between lesion and uterus; 3, sublet lesion with poorly defined edges; 4, well-seen lesion with poorly defined edges; 5, well-seen lesion with well-defined edges; Overall quality: 1, non-diagnostic; 2, substantial deficits in image quality; 3, moderate image quality; 4, good image quality; 5, excellent image quality) [17].

The same three radiologists independently assessed MR images to evaluate superficial (< 50% of myometrial depth) and deep myometraiao invasion (≥ 50% of myometrial depth) of EC. All readers were blinded to surgical histopathologic findings, but were told that the patients had been referred due to suspected EC. Patients were divided randomly into three groups. During one session, readers assessed the combination of rFOV-DWI and T2WI, or MUSE-DWI and T2WI, or DCE-MRI and T2WI. During other sessions, the same assessments were performed after switching the groups. The sessions were performed in three times, and were separated by a period of at least 2 weeks to minimize recall bias. Readers assessed superficial and deep myometrial invasion of EC using a 5-point scale: 1 = definitely absent, 2 = probably absent, 3 = equivocal, 4 = probably present, and 5 = definitely present. All readers were aware that a rating of 4 or 5 would be considered a positive diagnosis when calculating sensitivity and specificity. Before the first session, readers received following criteria: (a) DWI or DCE-MRI are main diagnostic sequence when evaluating myometrial invasion and T2WI provides a reference for assessing anatomic relations; (b) tumor is defined as a mass of high signal intensity (SI) relative to normal endometrium on high *b*-value (1000 s/mm^2^) DWI or a hypovascular mass compared to adjacent myometrium on DCE-MRI; (c) myometrial invasion is absent when a mass of high SI (DWI) or a hypovascular mass (DCE-MRI) is confined to the endometrial cavity; (d) superficial myometrial invasion is indicated when a high SI (DWI) or a hypovascular mass (DCE-MRI) extends to the inner half of the myometrium; and (e) deep myometrial invasion is indicated when high SI (DWI) or a hypovascular mass (DCE-MRI) extends to the outer half of the myometrium [13].

As a reference standard, a pathologist specializing in gynecologic pathology performed the following diagnostic process of myometrial invasion. The presence or absence of myometrial invasion of the tumor was first assessed, and when the myometrial invasion was present, then the deepest point of tumor invasion was determined and the depth of myometrial invasion was expressed as “less than half (superficial myometrial invasion)” or “half or more (deep myometrial invasion).

### Quantitative image analysis

Apparent diffusion coefficient (ADC) values were measured quantitatively by three radiologists (19, 10 and 5 years of experience in abdominal radiology). The average ADC values were calculated by drawing operator-defined regions of interest (ROIs) in EC and normal myometrium using a commercial image viewer (SYNAPSE SAI viewer; FUJIFILM). ROIs were placed on the ADC map of rFOV-DWI within the solid/homogeneous components and avoiding cystic/necrotic/inhomogeneous areas, and the ROIs were copied and pasted to MUSE ADC map. ROIs were placed at near-identical sites on both sequences and were as large as possible. The operators could refer to T2WI when placing ROIs.

The same ROIs were then copied and pasted into *b* = 1000 s/mm^2^ images. Average signal values within the ROI in EC and in normal myometrium were denoted as *S*_EC_ and *S*_M_, respectively. Signal-to-noise ratio (SNR) was defined as the average signal values (*S*_EC_ and *S*_M_) divided by standard deviation (SD) of each sequence (SD_EC_ and SD_M_, respectively) [17]:

$${\text{SNR}}_{{{\text{EC}}}} = S_{{{\text{EC}}}} /{\text{SD}}_{{{\text{EC}}}} ,\quad {\text{SNR}}_{{\text{M}}} = S_{{\text{M}}} /{\text{SD}}_{{\text{M}}}$$

Contrast-to-noise ratio (CNR) was defined as the absolute signal difference between EC and myometrium divided by the SD of the myometrium [17]:

$${\text{CNR}} = \left| {S_{{{\text{EC}}}} {-}S_{{\text{M}}} } \right|/{\text{SD}}_{{\text{M}}} .$$

### Statistical analysis

To evaluate the validity of the sample size, a post hoc power analysis was performed using G*Power software (latest ver. 3.1.9.6; Heinrich-Heine-Universität Düsseldorf, Germany; http://www.gpower.hhu.de/). A 2 by 2 Chi-square test was used to compare independent samples for the post hoc power analysis. The power (1 − *β*) was calculated from the effect size (w), *α*, and the total sample size (*n* = 55). A power (1 − *β*) of 0.8 or greater was considered the significance level [18]. Wilcoxon signed-rank test was used to assess the quality of images obtained with both DWI techniques. Inter-reader differences in sensitivity, specificity, and accuracy were compared using Cochran’s *Q* test. A receiver-operating characteristic (ROC) curve was fitted to each observer’s confidence rating. Median ADC, SNR, and CNR between rFOV and MUSE-DWI were compared using Mann–Whitney’s *U* test and Bland–Altman analysis. Inter-reader reliability was assessed by calculating intraclass correlation coefficients (ICCs). SPSS for Mac, version 24 (IBM, Chicago, USA) and JMP pro 16 (SAS Institute Inc, Cary, USA) were used for all statistical analyses. *p* value of < 0.05 was considered to indicate a statistically significant difference.

## Results

### Validity of sample size

The validity of the sample size was analyzed by post hoc power analysis for a group of 55 patients for whom the diagnostic performance of endometrial cancer was evaluated. When the minimum value of 0.61 was used as the effect size (w) and the *α* was set to 0.05, power (1 − *β*) was calculated to be 0.99. Therefore, the sample size of 55 in this study was a reasonable number of cases to assess the diagnostic performance of myometrial invasion.

### Surgical histologic findings

Histopathology confirmed that of the 58 tumors, 50 (86%) were endometrioid adenocarcinomas (grade 1, *n* = 39; grade 2, *n* = 10; grade 3, *n* = 1), three (5%) were serous adenocarcinomas, three (5%) were mixed cell carcinomas, and two (3%) were carcinosarcomas. Histologic examination also revealed that 11 patients (19%) had no myometrial invasion, 32 patients (55%) had superficial (< 50% of the myometrial depth), and 15 patients (26%) had deep myometrial invasion (≥ 50% of the myometrial depth).

### Image quality

**Fig. 2:**
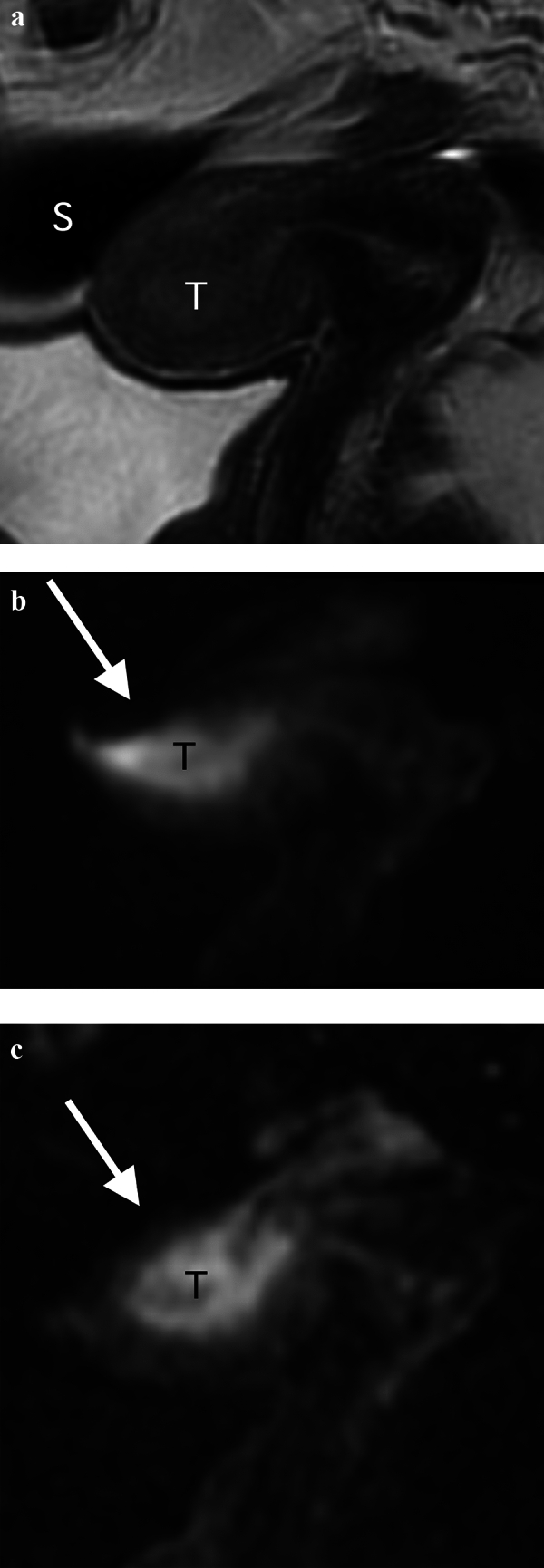
Images of a 66-year-old woman with endometrioid adenocarcinoma (Stage IB). **a** Parasagittal T2-weighted image shows a tumor (T) with intermediate signal intensity in the uterine cavity. There is gas-containing sigmoid colon (S) near the tumor. **b** Parasagittal reduced field-of-view diffusion-weighted image (rFOV-DWI) (*b* = 1000 s/mm^2^) shows the tumor (T) as an area of high signal intensity. Susceptibility artifacts are seen at air–tissue boundaries and the uterine structure is strongly distorted (arrow). **c** Parasagittal multiplexed sensitivity encoding diffusion-weighted image (MUSE-DWI) (*b* = 1000 s/mm^2^) shows the tumor (T) as an area of high signal intensity. MUSE-DWI depicts the tumor with significantly less susceptibility artifact compared to rFOV-DWI (arrow)

All three observers judged image quality was significantly better with MUSE-DWI than with rFOV-DWI in terms of artifacts (Fig. 2), sharpness, lesion conspicuity, and overall quality (all *p* < 0.05). There was no significant difference in noise between MUSE-DWI and rFOV-DWI among the observers (*p* = 0.18, 0.35, 0.53, respectively) (Table 1).

**Table 1:** Results of image quality scores

	MUSE-DWI > rFOV-DWI	MUSE-DWI = rFOV-DWI	MUSE-DWI < rFOV-DWI	Mean score of MUSE-DWI	Mean score of rFOV-DWI	*p*-value
Observer 1 (6 years of experience)						
Artifacts	21	30	7	3.48 ± 1.03	3.10 ± 0.87	0.002
Noise	7	38	13	3.38 ± 0.52	3.48 ± 0.54	0.18
Sharpness	23	28	7	3.98 ± 0.74	3.64 ± 0.81	0.002
Lesion conspicuity	20	33	5	4.24 ± 0.73	3.84 ± 0.79	0.001
Overall quality	20	31	7	3.78 ± 0.59	3.51 ± 0.60	0.009
Observer 2 (10 years of experience)						
Artifacts	38	19	1	4.41 ± 0.73	3.60 ± 0.72	< 0.0001
Noise	12	29	17	3.55 ± 0.50	3.64 ± 0.48	0.35
Sharpness	47	10	1	4.66 ± 0.58	3.67 ± 0.57	< 0.0001
Lesion conspicuity	49	9	0	4.76 ± 0.43	3.78 ± 0.50	< 0.0001
Overall quality	45	13	0	4.62 ± 0.52	3.67 ± 0.57	< 0.0001
Observer 3 (17 years of experience)						
Artifacts	23	24	11	3.59 ± 1.01	3.31 ± 0.86	0.018
Noise	15	29	14	3.33 ± 0.57	3.26 ± 0.71	0.53
Sharpness	36	22	0	3.64 ± 0.69	2.91 ± 0.66	< 0.0001
Lesion conspicuity	35	16	7	4.33 ± 0.69	4.17 ± 0.57	< 0.0001
Overall quality	30	22	6	3.64 ± 0.72	3.14 ± 0.80	< 0.0001

MUSE-DWI = Multiplexed sensitivity encoding diffusion-weighted imaging, rFOV-DWI = reduced field-of-view diffusion-weighted imaging

*p* values in square brackets indicate the results of statistical comparison of scores between MUSE and rFOV-DWI, calculated using the Wilcoxon signed-rank test

### Detection of myometrial invasion

Area under the curve (AUC), sensitivity, specificity, accuracy, positive predictive value (PPV), and negative predictive value (NPV) for myometrial invasion detection were assessed by the three observers.

**Fig. 3:**
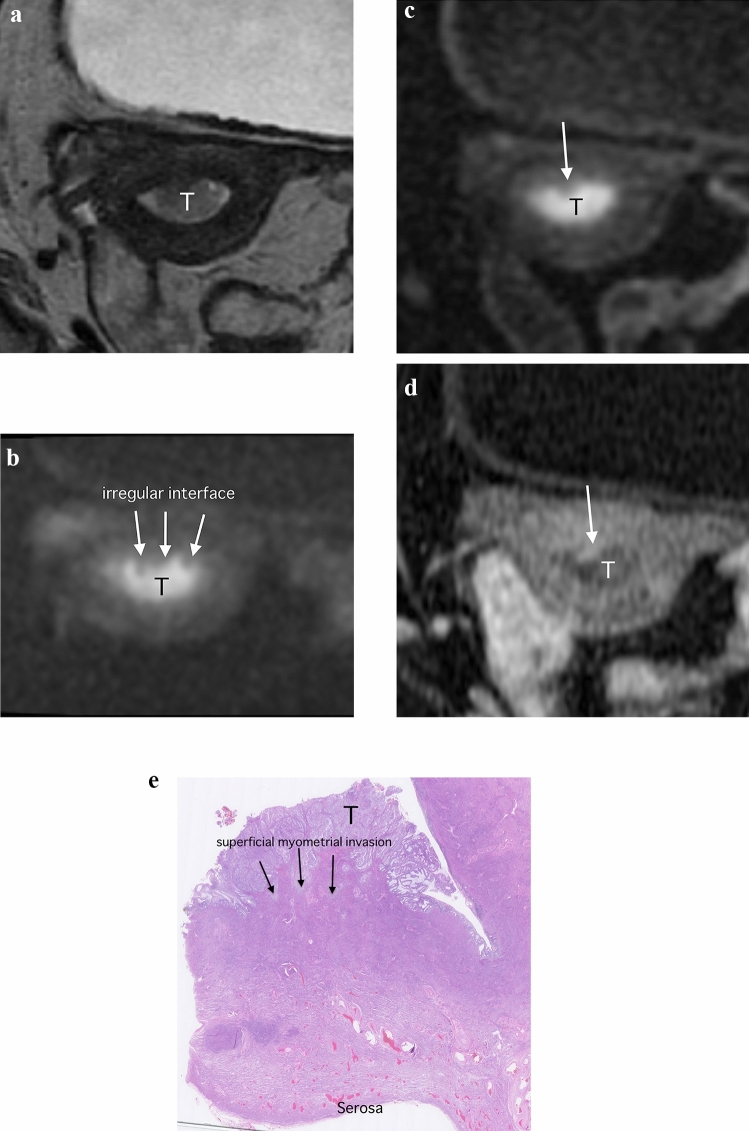
Images of a 61-year-old woman with endometrioid adenocarcinoma (Stage IA). **a** Para-axial T2-weighted image shows a tumor (T) with intermediate signal within the endometrial cavity. **b** Para-axial reduced field-of-view diffusion-weighted image (rFOV-DWI) (*b* = 1000 s/mm^2^) depicts the tumor (T) as an area of high signal intensity. The interface between the tumor and myometrium is irregular, suggesting myometrial invasion (arrow). **c** Para-axial multiplexed sensitivity encoding diffusion-weighted image (MUSE-DWI) (*b* = 1000 s/mm^2^) shows the tumor (T) as an area of high signal intensity, with high spatial resolution. However, the irregular interface between the tumor and myometrium is obscured (arrow). **d** Para-axial dynamic contrast-enhanced image (DCR-MRI) 2 min after contrast material administration shows the tumor (T) a hypovascular area. The irregular interface between the tumor and myometrium is also obscured. Surgical pathological findings confirmed the presence of superficial invasion, with absence of deep myometrial invasion. **e** Histopathologic image (hematoxylin–eosin stain; magnification, ×25) shows the tumor (T) invasion (arrows) into the inner half of the myometrium

Regarding superficial myometrial invasion, MUSE-DWI yielded significantly higher AUC value, sensitivity, and accuracy compared with DCE-MRI only in observer 1 (MUSE-DWI, 0.76 (95% CI 0.62–0.86), 72.7%, and 69.1% versus DCE-MRI, 0.64 (95% CI 0.50–0.76), 40.9%, and 50.9%; *p* = 0.049, 0.001 and < 0.001, respectively). Sensitivities were significantly higher with rFOV-DWI than with DCE-MRI in all observers (rFOV-DWI, 84.1%, 56.8% and 75.0% versus DCE-MRI, 40.9%, 36.4% and 52.3%; *p* ≤ 0.001, 0.018 and 0.005, respectively). Accuracies were also significantly higher with rFOV-DWI than with DCE-MRI in all observers (rFOV-DWI, 78.2%, 63.6% and 72.7% versus DCE-MRI, 50.9%, 49.1% and 60.0%; *p* ≤ 0.001, 0.009 and 0.001, respectively) (Table 2; Figs. 3 and 5).

**Table 2:** Diagnostic performances from an assessment of superficial and deep myometrial invasion

		AUC (95% CI)	Sensitivity (%)	Specificity (%)	Accuracy (%)	PPV (%)	NPV (%)
Observer 1 (6 years experiences)							
SMI	MUSE-DWI	0.76 (0.62–0.86)	72.7 (32/44)	54.5 (6/11)	69.1 (38/55)	86.5 (32/37)	33.3 (6/18)
	rFOV-DWI	0.74 (0.54–0.87)	84.1 (37/44)	54.5 (6/11)	78.2 (43/55)	88.1 (37/42)	46.2 (6/13)
	DCE-MRI	0.64 (0.50–0.76)	40.9 (18/44)	90.9 (10/11)	50.9 (28/55)	94.7 (18/19)	27.8 (10/36)
*p*-value	Between three		< 0.001*	0.069	< 0.001*	N/A	N/A
Bonferroni-adjusted *p*-value	MUSE-DWI versus rFOV-DWI	0.75	0.63	N/A	0.79	N/A	N/A
	MUSE-DWI versus DCE-MRI	0.049*	0.001*	N/A	< 0.001*	N/A	N/A
	rFOV-DWI versus DCE-MRI	0.31	< 0.001*	N/A	< 0.001*	N/A	N/A
DMI	MUSE-DWI	0.92 (0.82–0.97)	80.0 (12/15)	85.0 (34/40)	83.6 (46/55)	66.7 (12/18)	92.3 (34/37)
	rFOV-DWI	0.89 (0.75–0.96)	80.0 (12/15)	85.0 (34/40)	83.6 (46/55)	66.7 (12/18)	92.3 (34/37)
	DCE-MRI	0.80 (0.66–0.88)	20.0 (3/15)	100 (40/40)	78.2 (43/55)	100 (3/3)	76.9 (40/52)
*p*-value	Between three		< 0.001*	0.018*	< 0.001*	N/A	N/A
Bonferroni-adjusted *p*-value	MUSE-DWI versus rFOV-DWI	0.53	1.00	1.00	1.00	N/A	N/A
	MUSE-DWI versus DCE-MRI	0.022*	< 0.001*	0.043*	< 0.001*	N/A	N/A
	rFOV-DWI versus DCE-MRI	0.06	< 0.001*	0.043*	< 0.001*	N/A	N/A
Observer 2 (10 years experiences)							
SMI	MUSE-DWI	0.73 (0.55–0.86)	43.2 (19/44)	90.9 (10/11)	52.7 (29/55)	95.0 (19/20)	28.6 (10/35)
	rFOV-DWI	0.77 (0.59–0.89)	56.8 (25/44)	90.9 (10/11)	63.6 (35/55)	96.2 (25/26)	34.5 (10/29)
	DCE-MRI	0.76 (0.62–0.86)	36.4 (16/44)	100 (11/11)	49.1 (27/55)	100 (16/16)	28.2 (11/39)
*p*-value	Between three		0.019*	0.37	0.011*	N/A	N/A
Bonferroni-adjusted *p*-value	MUSE-DWI versus rFOV-DWI	0.48	0.20	N/A	0.70	N/A	N/A
	MUSE-DWI versus DCE-MRI	0.71	1.00	N/A	0.70	N/A	N/A
	rFOV-DWI versus DCE-MRI	0.89	0.018*	N/A	0.009*	N/A	N/A
DMI	MUSE-DWI	0.87 (0.70–0.95)	60.0 (9/15)	95.0 (38/40)	85.5 (47/55)	81.8 (9/11)	86.4 (38/44)
	rFOV-DWI	0.84 (0.69–0.93)	46.7 (7/15)	97.5 (39/40)	83.6 (46/55)	87.5 (7/8)	83.0 (39/47)
	DCE-MRI	0.82 (0.67–0.91)	46.7 (7/15)	97.5 (39/40)	83.6 (46/55)	87.5 (7/8)	83.0 (39/47)
*p*-value	Between three		0.64	0.78	0.50	N/A	N/A
Bonferroni-adjusted *p*-value	MUSE-DWI versus rFOV-DWI	0.71	N/A	N/A	N/A	N/A	N/A
	MUSE-DWI versus DCE-MRI	0.51	N/A	N/A	N/A	N/A	N/A
	rFOV-DWI versus DCE-MRI	0.78	N/A	N/A	N/A	N/A	N/A
Observer 3 (17 years experiences)							
SMI	MUSE-DWI	0.83 (0.67–0.92)	68.2 (30/44)	81.8 (9/11)	70.9 (39/55)	93.8 (30/32)	39.1 (9/24)
	rFOV-DWI	0.80 (0.62–0.91)	75.0 (33/44)	63.6 (7/11)	72.7 (40/55)	89.2 (33/37)	38.9 (7/18)
	DCE-MRI	0.80 (0.65–0.90)	52.3 (23/44)	90.9 (10/11)	60.0 (33/55)	95.8 (23/24)	32.3 (10/31)
*p*-value	Between three		0.005*	0.097	0.001*	N/A	N/A
Bonferroni-adjusted *p*-value	MUSE-DWI versus rFOV-DWI	0.63	1.00	N/A	0.45	N/A	N/A
	MUSE-DWI versus DCE-MRI	0.45	0.081	N/A	0.063	N/A	N/A
	rFOV-DWI versus DCE-MRI	0.96	0.005*	N/A	0.001*	N/A	N/A
DMI	MUSE-DWI	0.96 (0.86–0.99)	73.3 (11/15)	100 (40/40)	92.7 (51/55)	100 (11/11)	90.9 (40/44)
	rFOV-DWI	0.89 (0.77–0.95)	60.0 (9/15)	97.5 (39/40)	87.3 (48/55)	90.0 (9/10)	86.7 (39/45)
	DCE-MRI	0.95 (0.87–0.98)	53.3 (8/15)	97.5 (39/40)	85.5 (47/55)	88.9 (8/9)	84.8 (39/46)
*p*-value	Between three		0.37	0.37	0.69	N/A	N/A
Bonferroni-adjusted *p*-value	MUSE-DWI versus rFOV-DWI	0.048*	N/A	N/A	N/A	N/A	N/A
	MUSE-DWI versus DCE-MRI	0.73	N/A	N/A	N/A	N/A	N/A
	rFOV-DWI versus DCE-MRI	0.14	N/A	N/A	N/A	N/A	N/A

AUC = Area under the curve, SMI = superficial myometrial invasion, DMI = deep myometrial invasion, MUSE-DWI = multiplexed sensitivity encoding diffusion-weighted imaging, rFOV-DWI = reduced field-of-view diffusion-weighted imaging, DCE-MRI = dynamic contrast-enhanced magnetic resonance imaging, N/A = not applicable, * = statistically significant difference

**Fig. 4:**
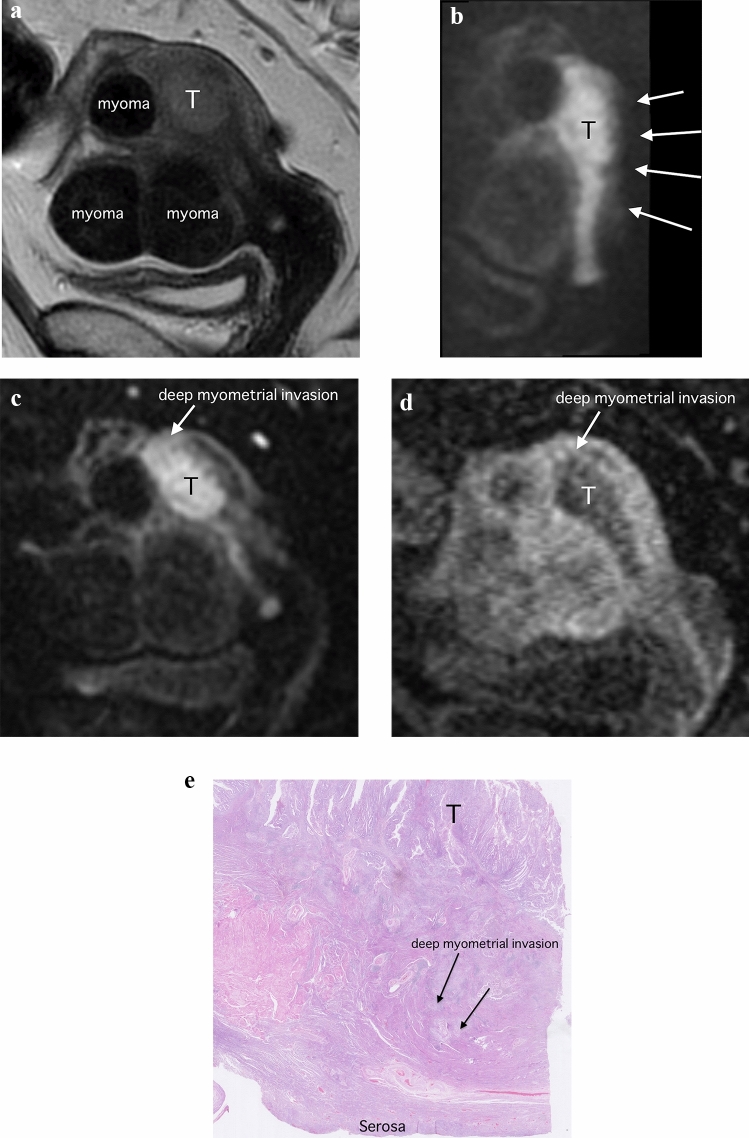
Images of a 56-year-old woman with endometrioid adenocarcinoma (Stage IIIC1). **a** Parasagittal T2-weighted image shows a tumor (T) with intermediate signal in the endometrium. **b** Parasagittal reduced field-of-view diffusion-weighted image (rFOV-DWI) (*b* = 1000 s/mm^2^) and **c** parasagittal multiplexed sensitivity encoding diffusion-weighted image (MUSE-DWI) (*b* = 1000 s/mm^2^) show the tumor (T) as an area of high signal intensity with extension into the myometrium (arrow). The edge of the uterus is blurred due to the small FOV on rFOV-DWI, so the deep myometrium is hard to see (arrows). In contrast, the entire uterine structure is well visualized on MUSE-DWI. **d** Parasagittal dynamic contrast-enhanced image (DCR-MRI) 2 min after contrast material administration shows the tumor (T) a hypovascular area. Deep myometrial invasion is seen (arrow). Pathological findings confirmed the presence of deep myometrial invasion. **e** Histopathologic image (hematoxylin–eosin stain; magnification, ×25) shows the tumor (T) invasion (arrows) into the outer half of the myometrium

**Fig. 5:**
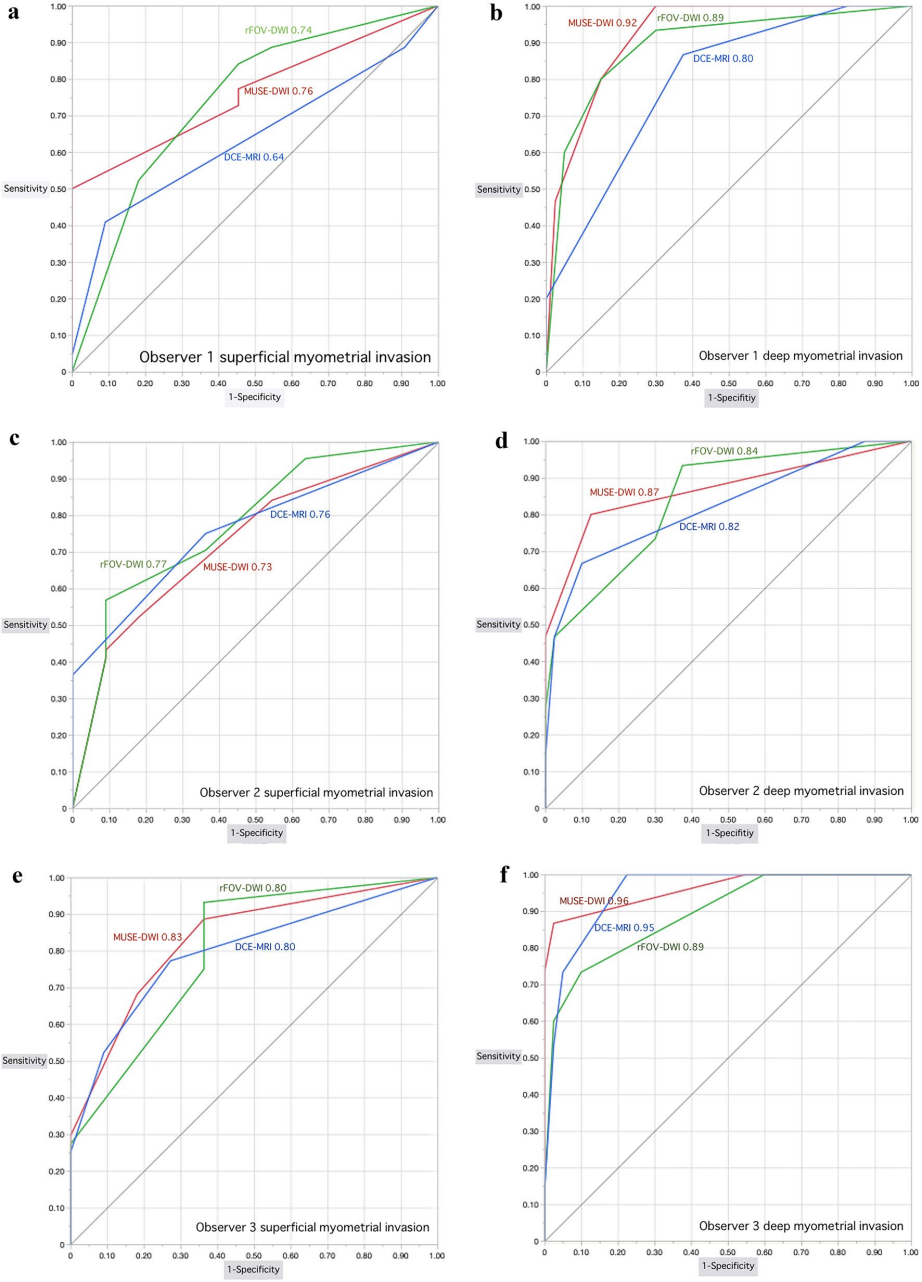
ROC curves of three observers. The red, green, and blue lines represent MUSE-DWI, rFOV-DWI, and DCE-MRI, respectively. **a** ROC curves on superficial myometrial invasion diagnosis for observer 1. **b** ROC curves on deep myometrial invasion for observer 1. **c** ROC curves on superficial myometrial invasion diagnosis for observer 2. **d** ROC curves on deep myometrial invasion for observer 2. **e** ROC curves on superficial myometrial invasion diagnosis for observer 3. **f** ROC curves on deep myometrial invasion for observer 3

Regarding deep myometrial invasion, MUSE-DWI yielded a significantly higher AUC value compared with DCE-MRI only in observer 1 (MUSE-DWI, 0.92 (95% CI 0.82–0.97) versus DCE-MRI, 0.80 (95% CI 0.66–0.88); *p* = 0.022). MUSE-DWI also showed significantly higher AUC compared with rFOV-DWI only in observer 3 (MUSE-DWI, 0.96 (95% CI 0.86–0.99) versus rFOV-DWI, 0.89 (95% CI 0.77–0.95); *p* = 0.048). MUSE-DWI and rFOV-DWI provided significantly higher sensitivities and accuracies (MUSE-DWI, 80.0% and 83.6%; rFOV-DWI, 80.0% and 83.6%) compared with those of DCE-MRI (20.0% and 78.2%; *p* < 0.001 and < 0.001, for sensitivites and accuracies, respectively) in observer 1. In observer 2 and 3, sensitivity, specificity, and accuracy were not significantly different between MUSE-DWI, rFOV-DWI, and DCE-MRI (Table 2; Figs. 4 and 5).

### Quantitative measurement

**Fig. 6:**
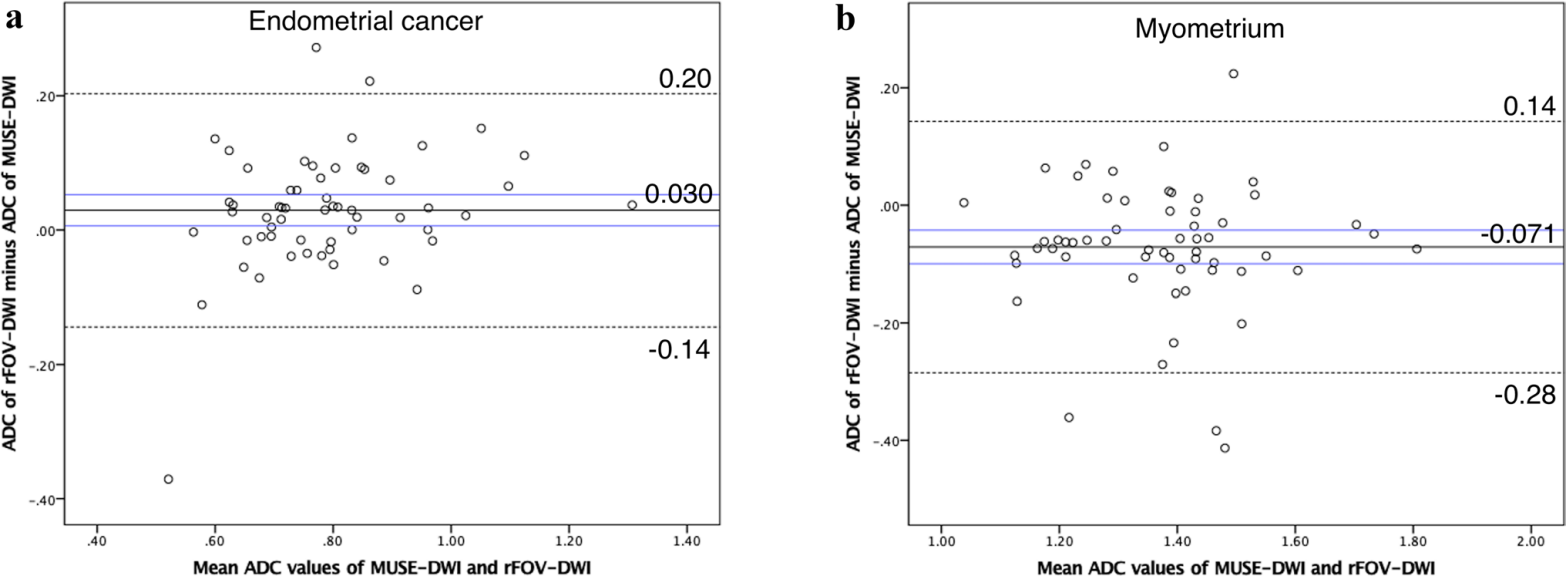
**a**, **b** Bland–Altman plots of average ADC values calculated from reduced field-of-view diffusion-weighted images (rFOV-DWI) and multiplexed sensitivity encoding diffusion-weighted images (MUSE-DWI) (*x*-axis) versus the ADC value difference between rFOV and MUSE-DWI (*y*-axis). The continuous line represents the mean absolute difference (bias) in ADC values between the two techniques; dashed lines represent the upper and lower limits of agreement; blue lines represent 95% confidence intervals of bias. Positive fixed bias is seen for tumor ADC values, and negative fixed bias is seen for myometrial ADC values

ADC_EC_ values measured by the three observers showed excellent reliability (ICC: MUSE-DWI = 0.80; rFOV-DWI = 0.75). The averaged median ADC_EC_ value on MUSE-DWI (0.76 × 10^−3^) was not significantly different compared to that on rFOV-DWI (0.79 × 10^−3^ mm^2^/s, *p* = 0.22). ADC_M_ values measured by the three observers showed fair to good reliability (MUSE-DWI = 0.59; rFOV-DWI = 0.63) (Table 3). The averaged median ADC_M_ value on MUSE-DWI (1.41 × 10^–3^) was significantly higher compared to that on rFOV-DWI (1.33 × 10^−3^ mm^2^/s, *p* = 0.022) (Table 4). In Bland–Altman plot of ADC_EC_ values, mean difference (rFOV-DWI – MUSE-DWI) was 0.030 × 10^−3^ mm^2^/s (95% confidence interval: 0.0061–0.053 × 10^−3^ mm^2^/s). Positive fixed bias was seen in ADC_EC_. The upper and lower limits of agreement were 0.20 and − 0.14 × 10^−3^ mm^2^/s (Fig. 6a). In Bland–Altman plot of ADC_M_ values, mean difference (rFOV-DWI–MUSE-DWI) was − 0.071 × 10^−3^ mm^2^/s (95% confidence interval: − 0.010 to − 0.042 × 10^−3^ mm^2^/s). Hence, negative fixed bias was also seen in ADC_M_. The upper and lower limits of agreement were 0.14 and − 0.28 × 10^−3^ mm^2^/s (Fig. 6b).

**Table 3:** Results of quantitative analysis (individual observer values)

	MUSE-DWI					rFOV-DWI				
	Observer 1	Observer 2	Observer 3	ICC	95% CI	Observer 1	Observer 2	Observer 3	ICC	95% CI
Median value of EC ADC (× 10^−3^ mm^2^/s)	0.77	0.73	0.75	0.80	0.69–0.88	0.82	0.75	0.79	0.75	0.61–0.85
IQR	0.69–0.87	0.66–0.83	0.68–0.84			0.71–0.93	0.67–0.88	0.70–0.87		
Median value of myometrium ADC (× 10^−3^ mm^2^/s)	1.35	1.41	1.41	0.59	0.43–0.72	1.31	1.37	1.33	0.63	0.49–0.75
IQR	1.23–1.45	1.28–1.53	1.31–1.60			1.16–1.42	1.23–1.47	1.21–1.47		
Median value of EC SNR	12.69	16.56	14.83	0.19	0.045–0.36	12.43	20.16	19.44	0.30	0.14–0.47
IQR	8.69–16.22	12.43–20.32	11.03–18.21			9.79–21.00	14.61–25.32	14.14–24.78		
Median value of myometrium SNR	8.93	8.78	9.60	0.41	0.25–0.57	10.68	11.95	13.21	0.10	–0.04–0.27
IQR	6.83–11.30	6.99–10.43	7.19–11.99			8.14–15.18	9.10–15.63	10.58–17.63		
Median value of CNR	14.06	15.91	14.64	0.63	0.50–0.75	14.23	17.02	19.84	0.50	0.33–0.64
IQR	10.83–20.32	11.05–21.08	11.71–22.13			9.56–20.01	11.96–26.91	14.30–25.14		

MUSE-DWI = Multiplexed sensitivity encoding diffusion-weighted imaging, rFOV-DWI = reduced field-of-view diffusion-weighted imaging, EC = endometrial cancer, ADC = apparent diffusion coefficient, SNR = signal to noise ratio, CNR = contrast to noise ratio, IQR = interquartile range, ICC = intraclass correlation coefficient, 95% CI = 95% confidence interval

**Table 4:** Results of quantitative analysis (average value of three observers)

	MUSE-DWI	rFOV-DWI	*p*-value
Median value of EC ADC (× 10^−3^ mm^2^/s)	0.76	0.79	0.22
IQR	0.69–0.83	0.70–0.90	
Median value of myometrium ADC (× 10^−3^ mm^2^/s)	1.41	1.33	0.022
IQR	1.26–1.51	1.23–1.42	
Median value of EC SNR	14.30	18.29	< 0.0001
IQR	11.63–17.34	14.25–21.36	
Median value of myometrium SNR	9.11	12.97	< 0.0001
IQR	7.72–10.52	10.46–15.29	
Median value of CNR	15.42	18.33	0.099
IQR	11.55–20.85	12.28–23.99	

MUSE-DWI = Multiplexed sensitivity encoding diffusion-weighted imaging, rFOV-DWI = reduced field-of-view diffusion-weighted imaging, EC = endometrial cancer, ADC = apparent diffusion coefficient, SNR = signal to noise ratio, CNR = contrast to noise ratio

IQR = interquartile range

*p* values indicate the results of statistical comparison of mean parameters (average of observer 1, 2 and 3) between MUSE and rFOV-DWI, calculated using the Mann–Whitney’s U test

SNR_EC_ values measured by the three observers showed poor reliability (MUSE-DWI = 0.19; rFOV-DWI = 0.30). Averaged SNR_EC_ of rFOV-DWI (median: 18.29) was significantly higher than that of MUSE-DWI (median: 14.30, *p* < 0.0001). SNR_EC_ values measured by the three observers showed poor to fair reliability (MUSE-DWI = 0.41; rFOV-DWI = 0.10). Averaged SNR_M_ of rFOV-DWI (median: 12.97) was also significantly higher than that of MUSE-DWI (median: 9.11, *p* < 0.0001). CNR values calculated by the three observers showed fair to good reliability (MUSE-DWI = 0.63; rFOV-DWI = 0.50) (Table 3). Averaged CNR of rFOV-DWI (median: 18.33) was not significantly different compared to that of MUSE-DWI (median: 15.42, *p* = 0.099) (Table 4).

## Discussion

Our study revealed superior overall image quality, less artifacts, increased image sharpness, and higher lesion conspicuity on MUSE-DWI than on rFOV-DWI. As for the diagnostic performance, the three observers showed inconsistent results and MUSE-DWI and rFOV-DWI provided almost equivalent diagnostic accuracies compared with DCE-MRI.cDWI enables rapid acquisition but is susceptible to artifacts such as image blurring, geometric distortion, chemical shift, and Nyquist ghosting. There is less distortion and ghosting with rFOV-DWI than with cDWI [12]. In EC, however, rFOV is clinically inadequate for evaluation of the whole pelvis (e.g., lymph node metastasis and peritoneal dissemination). Moreover, it has been reported that tumor ADC values obtained with rFOV-DWI are unstable [13, 19–23].

According to previous studies, image quality of both rFOV-DWI and MUSE-DWI is better compared with cDWI [13, 14, 17, 24–26]. In our study, MUSE-DWI was superior to rFOV-DWI in terms of artifacts, sharpness, lesion conspicuity, and overall quality. Therefore, MUSE technique may be more effective than rFOV technique in improving image quality of DWI. Contrary, there was no significant difference between the two sequences in terms of image noise. As MUSE-DWI is a time-consuming sequence, image noise cannot be improved due to limited NEX in MUSE-DWI (MUSE-DWI, 4; rFOV-DWI, 10).

Regarding the evaluation of superficial myometrial invasion, rFOV-DWI yielded significantly higher sensitivity and accuracy than DCE-MRI in all observers. Therefore, rFOV-DWI is considered to be a valuable additional sequence to DCE-MRI in clinical practice for assessing superficial myometrial invasion. The assessment of superficial myometrial invasion at MRI has a crucial role in patient selection for fertility-sparing treatment from the criteria for fertility-sparing treatment in EC [27]. We observed that the interface between the myometrium and tumor was better seen on rFOV-DWI than on MUSE-DWI in a few cases. rFOV-DWI may play an important role in the indication for fertility-preserving treatment due to its superior ability to depict the irregular interface between the tumor and myometrium. Although in-plane resolution in our study was similar between two sequences, rFOV might better delineate an irregular interface owing to its higher SNR and rectangular FOV with focus on the uterus. In a previous study, the accuracy of superficial myometrial invasion evaluation of rFOV-DWI was 48–64% [13], which is lower than that in the present study (accuracy, 64–77%). Due to lower in-plane-resolution of rFOV-DWI in our study than that in the previous study (previous study, 1.15 × 1.03; present study, 1.67 × 1.59 mm^2^), SNR of rFOV-DWI might have been higher in our study, and could be the reason for the favorable results. Differences in imaging planes used might also have affected diagnostic performance (previous study, para-axial plane only; present study, para-axial and parasagittal planes). With respect to AUC, MUSE-DWI showed significantly higher AUC than DCE-MRI in the most inexperienced radiologist. MUSE-DWI might increase the diagnostic confidence for the younger radiologist perhaps due to its higher spatial resolution and better image quality.

Regarding the evaluation of deep myometrial invasion, MUSE-DWI significantly improved the diagnostic performance (AUC, sensitivity, and accuracy) compared to DCE-MRI in one radiologist with the shortest experience. In the most experienced radiologists, the AUC of MUSE-DWI was significantly higher than that of rFOV-DWI. It might be easier to evaluate deep myometrial invasion with a wider FOV. Previous studies on deep myometrial invasion have reported accuracies of 82–98% with the combination of cDWI and T2WI, and of 84–92% with the combination of rFOV-DWI and T2WI [13, 28–32], which are in agreement with our result of combined rFOV-DWI and T2WI (83.6–87.3%).

Even though MUSE-DWI showed significantly higher image quality than rFOV-DWI, its diagnostic value was not consistent among the three observers. Nevertheless, we believe that the clinical utility of MUSE-DWI is promising. First, MUSE-DWI may be useful in the assessment of bulky tumor or enlarged uterus due to leiomyomas, because MUSE-DWI can provide a larger FOV. Indeed, in the present study, there were some cases that MUSE-DWI was superior to rFOV-DWI in depicting large uterus. Second, MUSE-DWI may be useful for assessment of extra-uterine diseases such as lymph node swellings or peritoneal nodules thanks to its large FOV. Since we did not evaluate the extra-uterine diseases, the value of MUSE-DWI might have been underestimated.

A comparison of ADC values between MUSE-DWI and rFOV-DWI shows inconsistent results. ADC values for EC were not significantly different between rFOV-DWI and MUSE-DWI, whereas those for myometrium were significantly higher on MUSE-DWI than on rFOV-DWI. Bland–Altman analyses showed ADC values for EC tended to be higher on rFOV-DWI than on MUSE-DWI, whereas ADC values for myometrium tended to be higher on MUSE-DWI than on rFOV-DWI. Previous studies have reported no significant difference in ADC values between MUSE-DWI and cDWI in the liver, female pelvis, and breast [17, 24, 26]. ADC values are affected by various parameters such as TR and TE, as well as image noise [33]. In our study, differences in TR/TE between two sequences (rFOV-DWI, 4500/66.2; MUSE-DWI, 6500/78.7 ms) as well differences in SNR might explain the inconsistent results. Nevertheless, the difference in ADC values is small and may not be a clinical problem.

SNR obtained with rFOV-DWI was significantly higher than that with MUSE-DWI despite the similar spatial resolution of the two sequences. This finding is in agreement with that of previous study that reported lower SNR with MUSE-DWI than cDWI due to differences in spatial resolution between the sequences [17]. We consider that higher NEX applied for rFOV-DWI was the main contributor to its higher SNR compared with MUSE-DWI. As MUSE-DWI is already a lengthy sequence, we cannot increase NEX in clinical practice.

Our study has some limitations. First, this was a single center retrospective study with relatively small sample size. However, from the post hoc power analysis, the sample size of this study was considered to be adequate. Second, cDWI was not assessed, because our main purpose was to compare diagnostic performance and image quality between MUSE-DWI and rFOV-DWI. Third, 8 of 58 tumors were other than endometrioid adenocarcinoma. The image findings of myometrial invasion in these tumors might be different from those of endometrioid adenocarcinoma. Forth, in the present study, we evaluated only the equilibrium phase images for DCE-MRI. This is because tumor-to-myometrium contrast is the best at this timing [34]. Smooth and clear subendometrial enhancement (SEE), which is best seen approximately 35–45 s after contrast injection, is reported to indicate the absence of myometrial invasion on DCE-MRI [35]. Since we did not assess SEE in the current study, the diagnostic capacity of DCE-MRI for assessment of superficial myometrial invasion might have been lowered.

In conclusion, MUSE-DWI showed significantly better image quality than rFOV-DWI in the female pelvis. MUSE-DWI and rFOV-DWI showed almost the same diagnostic performance compared to DCE-MRI regarding superficial and deep myometrial invasion of EC although MUSE-DWI may be helpful for some radiologists.

## Abbreviations

T2WI: T2-weighted imaging
DCE: Dynamic contrast-enhanced
EC: Endometrial cancer
DWI: Diffusion-weighted imaging
cDWI: Conventional diffusion-weighted imaging
EPI: Echo-planar imaging
rFOV: Reduced field-of-view
FOV: Field-of-view
MUSE: Multiplexed sensitivity encoding
MUSE-DWI: Multiplexed sensitivity encoding diffusion-weighted imaging
rFOV-DWI: Reduced field-of-view diffusion-weighted imaging
NEX: Number of excitations
SI: Signal intensity
ADC: Apparent diffusion coefficient
ROI: Region of interest
SNR: Signal-to-noise ratio
SD: Standard deviation
CNR: Contrast-to-noise ratio
ROC: Receiver operating characteristic
ICC: Intraclass correlation coefficients
AUC: Area under the curve
PPV: Positive predictive value
NPV: Negative predictive value

## Declarations

### Guarantor

The scientific guarantor of this publication is Takashi Ota.

### Statistics and biometry

Takahiro Tsuboyama kindly provided statistical advice for this manuscript. One of the authors has significant statistical expertise.

### Study subjects or cohorts overlap

Study subjects (humans) or cohorts have not been previously reported.

### Methodology

Retrospective, diagnostic or prognostic study, performed at one institution.
