# Correction: Diagnostic accuracy of MRI for evaluating myometrial invasion in endometrial cancer: a comparison of MUSE-DWI, rFOV-DWI, and DCE-MRI

**DOI:** 10.1007/s11547-024-01928-2

**Published:** 2024-11-16

**Authors:** Takashi Ota, Takahiro Tsuboyama, Hiromitsu Onishi, Atsushi Nakamoto, Hideyuki Fukui, Keigo Yano, Toru Honda, Kengo Kiso, Mitsuaki Tatsumi, Noriyuki Tomiyama

**Affiliations:** Department of Diagnostic and Interventional Radiology, Osaka University Graduate School of Medicine, D1, 2-2, Yamadaoka, Suita, Osaka 565-0871 Japan

**Correction to: La radiologia medica (2023) 128:629** 10.1007/s11547-023-01635-4

The article Diagnostic accuracy of MRI for evaluating myometrial invasion in endometrial cancer: a comparison of MUSE-DWI, rFOV-DWI, and DCE-MRI, written by Takashi Ota, Takahiro Tsuboyama, Hiromitsu Onishi, Atsushi Nakamoto, Hideyuki Fukui, Keigo Yano, Toru Honda, Kengo Kiso, Mitsuaki Tatsumi, Noriyuki Tomiyama, was originally published Online First without Open Access. After publication in volume 128, issue 6, page 629–643, CRUE-CSIC consortium requested that the article be Open Choice to make the article an open access publication. Open access funding is provided by Misión Biológica de Galicia (MBG-CSIC), within the CRUE-CSIC Agreement. Therefore, the copyright of the article has been changed to © The Author(s) 2024 and the article is forthwith distributed under the terms of the “Creative Commons Attribution 4.0 International License, which permits use, sharing, adaptation, distribution and reproduction in any medium or format, as long as you give appropriate credit to the original author(s) and the source, provide a link to the Creative Commons licence, and indicate if changes were made. The images or other third-party material in this article are included in the article’s Creative Commons licence, unless indicated otherwise in a credit line to the material. If material is not included in the article’s Creative Commons licence and your intended use is not permitted by statutory regulation or exceeds the permitted use, you will need to obtain permission directly from the copyright holder. To view a copy of this licence, visit http://creativecommons.org/licenses/by/4.0/”.

Funding information section was missing from this article and should have read "Funding Note: **Open Access funding provided by Osaka University**".

The original article has been corrected.

